# Repeated intermittent hypoxic stimuli to operative lung reduce hypoxemia during subsequent one-lung ventilation for thoracoscopic surgery: A randomized controlled trial

**DOI:** 10.1371/journal.pone.0249880

**Published:** 2021-04-15

**Authors:** Susie Yoon, Bo Rim Kim, Se-Hee Min, Jaehun Lee, Jae-Hyon Bahk, Jeong-Hwa Seo

**Affiliations:** 1 Department of Anesthesiology and Pain Medicine, Seoul National University Hospital, Seoul National University College of Medicine, Seoul, Republic of Korea; 2 Department of Anesthesiology and Pain Medicine, Chung-Ang University College of Medicine, Seoul, Republic of Korea; Prince Sattam Bin Abdulaziz University, College of Applied Medical Sciences, SAUDI ARABIA

## Abstract

**Background:**

An intervention to potentiate hypoxic pulmonary vasoconstriction may reduce intrapulmonary shunt and hypoxemia during one-lung ventilation. Previous animal studies reported that repeated intermittent hypoxic stimuli potentiated hypoxic pulmonary vasoconstriction, but no clinical study has examined the effects of this intervention on hypoxemia during one-lung ventilation. We thus performed a single-center, parallel-group, double-blind, randomized controlled trial to investigate whether repeated intermittent hypoxic stimuli to the operative lung reduce hypoxemia during the subsequent one-lung ventilation for thoracoscopic surgery.

**Methods:**

Patients undergoing one-lung ventilation were randomized into two groups (n = 68 each). Before one-lung ventilation, in the intermittent hypoxia group, the nondependent lung was not ventilated for 2 min and then ventilated for 2 min while the dependent lung was continuously ventilated. This was repeated five times. In the continuous normoxia group, both lungs were ventilated for 20 min. We measured SpO_2_, PaO_2_, FiO_2_, PaCO_2_, SaO_2_, and central venous oxygen saturation during one-lung ventilation. The primary outcome was the number of patients with hypoxemia defined as a SpO_2_ <95% during one-lung ventilation, which was analyzed with a chi-squared test.

**Results:**

Hypoxemia was less frequent in the intermittent hypoxia group than in the continuous normoxia group during OLV [6/68 (8.8%) vs 17/68 (25.0%), risk ratio (95% CI) 0.35 (0.15–0.84), p = 0.012]. The PaO_2_ (p = 0.008 for 30 min and 0.007 for 60 min) and PaO_2_/FiO_2_ (p = 0.008 for both) were higher 30 and 60 min after starting one-lung ventilation, and the alveolar-arterial pressure gradient (p = 0.010) and shunt index (p = 0.008) were lower 30 min after starting one-lung ventilation in the intermittent hypoxia group than in the continuous normoxia group. Postoperative adverse events did not differ significantly between groups.

**Conclusions:**

Repeated intermittent hypoxic stimuli to the operative lung seemed to potentiate hypoxic pulmonary vasoconstriction, and thus reduced hypoxemia during the subsequent one-lung ventilation.

## Introduction

To improve the surgical conditions for pulmonary resection, the operative lung is deflated while the contralateral lung is ventilated. During the one-lung ventilation (OLV), because the deflated lung is still perfused, the intrapulmonary shunt impairs arterial oxygenation [1–4]. However, alveolar hypoxia developed in the deflated lung leads to the constriction of the ipsilateral resistance pulmonary arteries [5–9]. This hypoxic pulmonary vasoconstriction (HPV) diverts blood flow from the non-ventilated lung to the ventilated lung, minimizing ventilation-perfusion mismatch and improving arterial oxygenation [5–7, 9–11]. Therefore, an intervention to potentiate HPV may reduce the intrapulmonary shunt and hypoxemia during OLV [12–15].

Several animal studies reported that repeated intermittent hypoxic stimuli potentiated the subsequent HPV [12–15]. This intervention was also used in an observational pediatric study [16], a non-randomized study about biomarkers of lung injury [17], and a randomized study that only examined a PaO_2_/FiO_2_ 30 min after OLV and postoperative outcomes [18]. However, no study has examined the effects of this intervention on intraoperative hypoxemia, even though 7–48% of thoracic surgical patients show hypoxemia during OLV [2, 10, 19–21]. We thus conducted a prospective randomized trial to test the hypothesis that repeated intermittent hypoxic stimuli to the operative lung reduce hypoxemia during the subsequent OLV for thoracoscopic surgery in the lateral decubitus position.

## Materials and methods

### Patients

This prospective, single-center, parallel-group, double-blind, randomized controlled trial was approved by the Institutional Review Board (IRB #1705-090-855) of Seoul National University Hospital (Seoul, Korea) and was registered prior to patient enrollment at ClinicalTrials.gov (NCT03282032, Principal investigator: Jeong-Hwa Seo, Date of registration: 8 September 2017). After obtaining written informed consent, we enrolled patients aged 20–70 yr with ASA physical status I–III, and undergoing elective thoracoscopic surgery under OLV using a left-sided double-lumen tube in the lateral decubitus position between November 2017 and October 2018. We excluded patients with severe cardiopulmonary diseases, history of pulmonary resection, planned bilateral lung surgery, irregular cardiac rhythm, anemia, pregnancy, and body mass index >35 kg m^-2^.

Patients were randomized into two groups to receive five intermittent hypoxic stimuli (intermittent hypoxia group) or one continuous normoxic stimulus (continuous normoxia group) to the operative lung before OLV. An assistant unrelated to the study assigned the group in a 1:1 ratio using a complete randomization procedure without stratification, blocking, and terminal balance, and concealed the allocation sequence in serially-numbered, sealed, and opaque envelopes. After induction of general anesthesia, the attending anesthesiologist, who was not the investigator of this study, opened the envelope and conducted the experimental intervention according to the group assignment. Investigators did not enter the operating room during the experimental protocol for blinding of group assignment. The manuscript adheres to the applicable Consolidated Standards of Reporting Trials guidelines. No changes to the protocol are made after trial commencement, and the trial ended after recruiting all participants without interim analyses. The trial protocol is provided in the (S1 and S2 Protocols).

### Anesthesia and experimental protocol

Patients were monitored with non-invasive blood pressure, pulse oximetry, electrocardiography, bispectral index (A-2000 XP; Aspect Medical Systems, Newton, MA, USA), and acceleromyography (TOF-watch Sx; Organon, Dublin, Ireland). Propofol (Fresofol MCT 2%; Fresenius Kabi, Homburg, Germany) and remifentanil (Ultiva; GlaxoSmithKline, Brentford, Middlesex, UK) were administered intravenously with effect-site target-controlled infusion (Orchestra; Fresenius Kabi, Brézins, France). The initial target concentration was 3–5 μg ml^-1^ for propofol, and 3–5 ng ml^-1^ for remifentanil.

After intravenous administration of rocuronium 0.6–0.8 mg kg^-1^, train-of-four (TOF) counts were monitored at the adductor pollicis muscle every 15 s. At a TOF count = 0 and bispectral index <60, a double-lumen tube (Mallinckrodt endobronchial tube; Covidien, Mansfield, MA, USA) was placed into the left mainstem bronchus by direct or video laryngoscopy. The 32, 35, 37, or 39-Fr double-lumen tubes were selected based on the left bronchial diameter measured on the preoperative chest computed tomography, or sex and height of the patient.

With fiberoptic bronchoscopy (LF-DP or LF-GP; Olympus Optical Co., Tokyo, Japan), we positioned the bronchial cuff of the double-lumen tube into the left bronchus below the carina without herniation, and the bronchial tip above the left lobar bronchi without obstruction. If the tube was malpositioned into the right bronchus, it was repositioned into the left bronchus with bronchoscopic guidance. The tracheal and bronchial cuff pressures were adjusted to <25 cm H_2_O using a portable manometer (VBM Medizintechnik GmbH, Sulz am Neckar, Germany).

A 20-gauge catheter was inserted into the radial artery and was connected to a transducer for arterial waveform analysis (FloTrac, version 4.0; Edwards Life Sciences, Irvine, CA, USA). An 8.5-Fr central venous oximetry catheter (PreSep; Edwards Lifesciences, Irvine, CA, USA) was inserted into the right internal jugular vein, and its tip was positioned at the junction of the superior vena cava and right atrium with sonographic guidance (Vivid i; GE Healthcare, Chicago, IL, USA) [22]. The PreSep catheter was calibrated in vivo with hemoglobin, hematocrit, and oxygen saturation (ScvO_2_) values measured in the central venous blood. The arterial and venous pressure transducers were placed at the right atrium level and were exposed to atmospheric pressure for zeroing.

After moving the patient to the lateral decubitus position during surgical preparation, we adjusted the positions of the double-lumen tube and pressure transducers. In the intermittent hypoxia group, the nondependent lung was not ventilated for 2 min and then ventilated for 2 min while the dependent lung was continuously ventilated (S1 Fig). The non-ventilation and ventilation of the nondependent lung was repeated five times. In the continuous normoxia group, both lungs were ventilated for 20 min. In both groups, the anesthetic machine (Primus; Dräger, Lübeck, Germany) was set to an FiO_2_ 1.0 and tidal volume 6 ml kg^-1^ of predicted body weight (PBW) during non-ventilation of the dependent lung, and to an FiO_2_ 0.5 and tidal volume 8 ml kg^-1^ of PBW during ventilation of both lungs. The PBW was calculated as 50.0+0.905×(height-152.4) for men, and 45.5+0.905×(height-152.4) for women [23]. A respiratory rate 12 breaths min^-1^, PEEP 5 cm H_2_O, inspiratory: expiratory (I:E) ratio 1:2, and fresh gas flow 2 liter min^-1^ were applied during the experimental protocol in both groups.

After the protocol, OLV (defined as the ventilation of only the dependent lung before or after starting operation) was initiated with an FiO_2_ 0.8, PEEP 5 cm H_2_O, I:E ratio 1:2, tidal volume 4–8 ml kg^-1^ of PBW, and respiratory rate 12–20 breaths min^-1^ to maintain an SpO_2_ ≥95%, PaO_2_ 13.3–33.3 kPa (100–250 mm Hg), PaCO_2_ 4.7–6.7 kPa (35–50 mm Hg), and peak inspiratory pressure <30 cm H_2_O. When a PaO_2_ was ≥33.3 kPa (250 mm Hg), the FiO_2_ was decreased to 0.6; and when a PaO_2_ was <13.3 kPa (100 mm Hg), the FiO_2_ and PEEP were increased to 1.0 and 8 mm Hg, respectively. If SpO_2_ decreased to <95%, we applied alveolar recruitment maneuvers to the dependent ventilated lung with an FiO_2_ 1.0, end-inspiratory pressure 30 cm H_2_O, PEEP 10 cm H_2_O, and I:E ratio 1:1 until SpO_2_ increased to ≥98% after checking the double-lumen tube position with fiberoptic bronchoscopy. If SpO_2_ dropped to <90%, we asked the surgeon to stop the operation, and ventilated both lungs manually with an FiO_2_ 1.0 until SpO_2_ increased to ≥98%. The effect-site concentrations of propofol and remifentanil were titrated for a bispectral index 30–60, and rocuronium 0.2–0.3 mg kg^-1^ was intermittently administered for a TOF count = 0. Ephedrine 5–10 mg, phenylephrine 30–50 μg, or plasmalyte 100–200 ml were administered intravenously to manage a mean blood pressure <60 mm Hg, central venous pressure <4 mm Hg, stroke volume variation >13%, or urine output <0.5 ml kg^-1^ h^-1^ according to the attending anesthesiologist’s discretion, when one or more conditions were met. A packed red blood cell was transfused to maintain a hematocrit level >20%.

After pulmonary resection, the nondependent operative lung was inflated and then bilateral lungs were ventilated with an FiO_2_ 0.5. A chest tube was inserted into the operative hemithorax through the incision for the thoracoscopic port. Intravenous patient-controlled analgesia (Automed 3200; Ace Medical, Seoul, Korea) was started with a 100-ml mixture of fentanyl 1000–2000 μg, morphine 40–80 mg, ramosetron 0.3–0.6 mg, and saline at an infusion of 1 ml h^-1^, bolus of 0.5 ml, and lockout time of 10 min.

After surgery, patients were turned to the supine position and secretions were suctioned from both lungs. The double-lumen tube was removed after administration of sugammadex 4 mg kg^-1^ when the patient had spontaneous breathing, responses to verbal commands, and a TOF ratio >0.9. If mechanical ventilation was required after surgery, the double-lumen tube was replaced with a plain tube without the administration of sugammadex. Patients were transferred to post-anesthesia or intensive care units.

### Outcomes

We collected data for characteristics of patients, surgery, and anesthesia. We continuously monitored SpO_2_ during OLV and defined mild hypoxemia as 90%≤ SpO_2_ <95% and severe hypoxemia as SpO_2_ <90% [2, 3, 7, 10, 21, 24–27]. During OLV, if patients experienced more than one episode of mild hypoxemia or severe hypoxemia, we recorded the time of the first episode. If patients had both mild and severe hypoxemia, it was counted as severe hypoxemia. Immediately before and 30, 60, 90, and 120 min after starting OLV, we measured PaO_2_, PaCO_2_, SaO_2_, and hematocrit with a blood gas analyzer (GEM premier 3000, Model 5700; Instrumentation Laboratory, Lexington, MA, USA); FiO_2_, PEEP, I:E ratio, EtCO_2_, tidal volume, respiratory rate, and peak or plateau airway pressures, with the Primus anesthetic machine; mean arterial pressure, heart rate, and cardiac index with the FloTrac sensor; and ScvO_2_ with the PreSep catheter. Some variables were calculated as follows: PaO_2_/FiO_2_; alveolar-arterial oxygen pressure gradient [P(A-a)O_2_] = 95×FiO_2_-PaCO_2_/0.8-PaO_2_ [6, 9, 27, 28]; shunt index = (100-SaO_2_)/(100-ScvO_2_) ×100 [29]; minute ventilation = tidal volume×respiratory rate; alveolar dead space = tidal volume× (PaCO_2_-EtCO_2_)/PaCO_2_ [30]; static pulmonary compliance = tidal volume/(plateau pressure-PEEP) [31]; and dynamic pulmonary compliance = tidal volume/(peak inspiratory pressure-PEEP) [31].

At 30 min after surgery, PaO_2_, PaCO_2_, and hematocrit were checked while oxygen was supplied via a facemask at a rate of 5 liter min^-1^. The chest tube was removed when the drainage was less than 200–250 ml, and no air leak was detected. We recorded lengths of stay in the post-anesthesia or intensive care units and in the hospital. Patients were discharged from the post-anesthesia care unit when the modified Aldrete score was ≥9; from the intensive care unit when they had no cardiorespiratory problems and did not require inotropes or oxygen therapy; and from the hospital when they were able to ambulate, cough, and clear sputum without severe pain, fever, and complications. We recorded any perioperative adverse events.

The primary outcome was the number of patients with SpO_2_ <95% during the entire period of OLV. Secondary outcomes were the number of episodes and the number of patients with mild or severe hypoxemia during OLV; PaO_2_, PaO_2_/FiO_2_, P(A-a)O_2_, shunt index, and respiratory or hemodynamic variables including minute ventilation, alveolar dead space, static and dynamic pulmonary compliances, FiO_2_, PEEP, I:E ratio, PaCO_2_, hematocrit, mean arterial pressure, heart rate, and cardiac index immediately before (baseline) and 30, 60, 90, and 120 min after starting OLV; duration of the chest tube drainage and lengths of stay in the postoperative care units and hospital after surgery; and perioperative adverse events.

### Statistical analysis

Continuous variables were summarized as mean (SD) and were compared with unpaired t-tests (duration of the chest tube drainage; and lengths of stay in the postoperative care units and hospital after surgery). Repeatedly measured longitudinal data were analyzed with linear mixed effects models (PaO_2_, PaO_2_/FiO_2_, P(A-a)O_2_, shunt index, and respiratory or hemodynamic variables before and 30, 60, 90, and 120 min after starting OLV). A first-order autoregressive correlation structure was assumed to model the within-subject correlation over time. The fixed effects were the time (as a categorical variable), group, and interaction between the time and group. If the interaction was significant, pairwise comparisons between groups were performed at each time point. If no interaction was detected, the main effect collapsed over time was reported. In within-group comparisons, the measurement at each time point after starting OLV was compared with the baseline (before OLV).

Categorical variables were presented as the number of patients and were compared with a chi-squared test (number of patients with SpO_2_ <95% during OLV, primary outcome) or Fisher’s exact tests (number of patients with mild or severe hypoxemia during OLV, and perioperative adverse events).

All the statistical analyses were two-sided and a significance criterion was P <0.05. Risk ratios or mean differences with 95% CIs were calculated for the primary outcome and secondary outcomes as appropriate. All analyses were conducted in an intention-to-treat manner. STATA (Special Edition 14.2; Stata Corporation, College Station, Texas, USA) was used for statistical comparisons between and within groups, power analyses, and group randomization.

In our pilot study (n = 10), three patients (30%) showed hypoxemia with SpO_2_ <95% during OLV when no pretreatment had been applied before OLV. To detect a 20% difference in the number of hypoxemic patients using a chi-squared test, 62 patients were needed in each group with an α of 0.05 and power of 0.8 for two-sided analysis. We planned to enroll 68 patients in each group considering the dropout rate of 10%.

## Results

**Fig 1:**
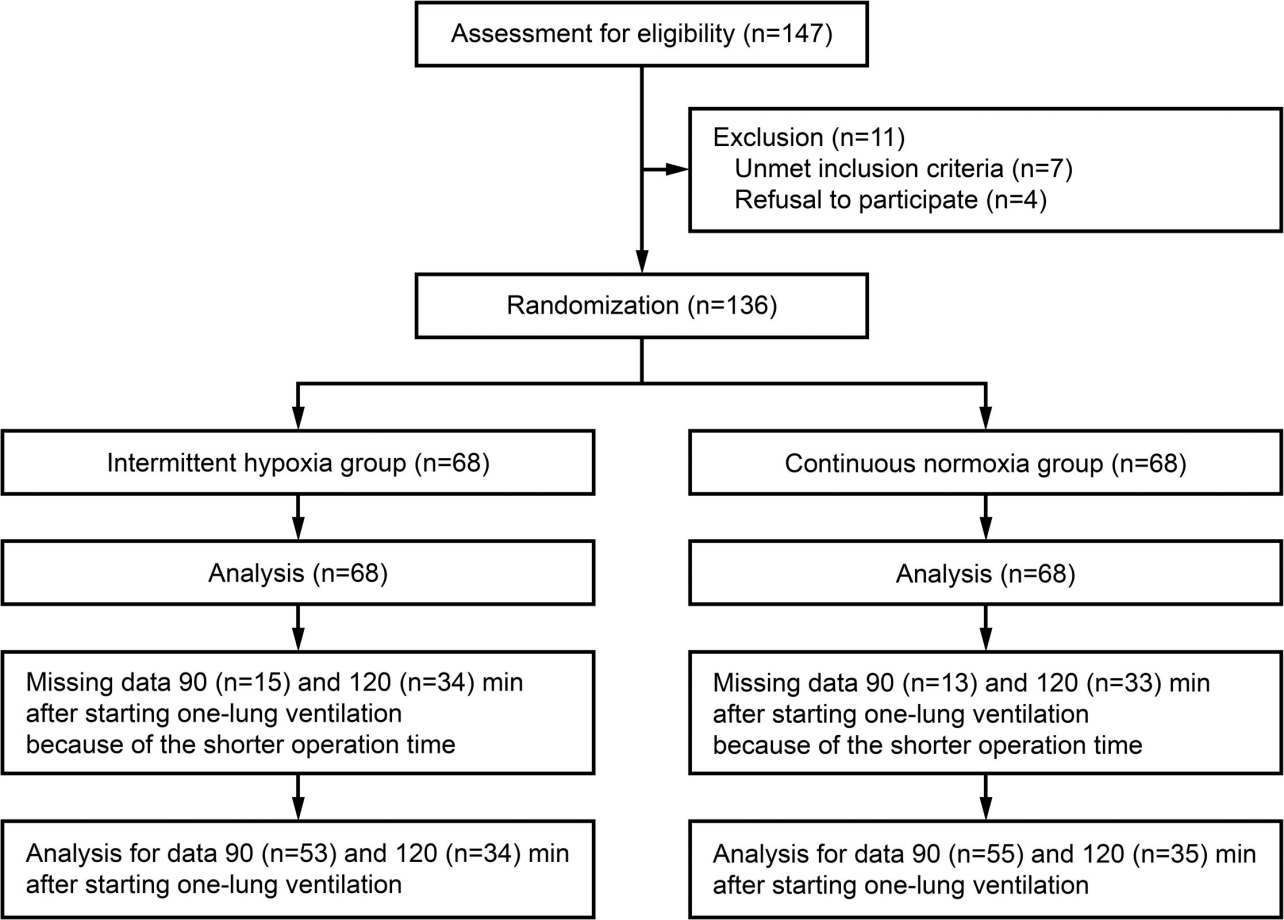
CONSORT diagram.

After screening 147 patients, 136 were randomized to the intermittent hypoxia or continuous normoxia groups (n = 68 each; Fig 1). All the 136 patients were included for all the statistical analyses, but some data were missing 90 and 120 min after starting OLV because of the shorter operation time (Fig 1). No clinically relevant differences were observed between groups in patient characteristics (Table 1).

**Table 1:** Characteristics of patients, surgery, and anesthesia.

	Intermittent hypoxia group (n = 68)	Continuous normoxia group (n = 68)
Age	60 (8)	59 (10)
Female	37	38
Height (cm)	162 (9)	162 (9)
Weight (kg)	63 (10)	62 (10)
Predicted body weight (kg)	56 (10)	56 (10)
Body mass index (kg m^-2^)	24.1 (3.0)	23.8 (3.1)
ASA physical status (II/III)	25/8	26/6
Diagnosis (malignancy/benign)	58/10	62/6
Comorbidity (hypertension/diabetes/angina/stroke/hepatitis)	15/10/4/3/1	16/10/4/2/1
Smoker	12	11
Forced expiratory volume in 1 s (liter)	2.6 (0.6)	2.6 (0.7)
Forced vital capacity (liter)	3.5 (0.8)	3.4 (0.9)
Forced expiratory volume in 1 s/Forced vital capacity (%)	75.3 (7.3)	77.0 (7.6)
Diffusing capacity of lung for carbon monoxide (ml kPa^-1^ min^-1^)	17.9 (4.3)	18.0 (4.4)
PaO_2_ (kPa, room air)	13.6 (1.8)	13.7 (2.5)
PaCO_2_ (kPa, room air)	5.4 (0.5)	5.4 (0.5)
Hematocrit (%)	39.9 (4.1)	39.2 (3.1)
Thoracoscopic procedure (wedge resection/segmentectomy/lobectomy)	5/6/57	4/6/58
Side of surgery (left/right)	19/49	25/43
Amount of anesthetic drugs and a fluid		
Propofol (mg)	1184 (358)	1257 (438)
Remifentanil (μg)	1383 (421)	1461 (531)
Rocuronium (mg)	100 (18)	103 (27)
Plasmalyte (ml)	602 (286)	584 (267)
Inotropic requirement	22	21
Estimated blood loss (ml)	89 (77)	81 (68)
Duration of intervention		
One-lung ventilation (min)	105 (35)	111 (41)
Surgery (min)	131 (43)	133 (50)
Anesthesia (min)	182 (41)	186 (53)

Data are mean (standard deviation) or number of patients. ASA, American Society of Anesthesiologists.

**Fig 2:**
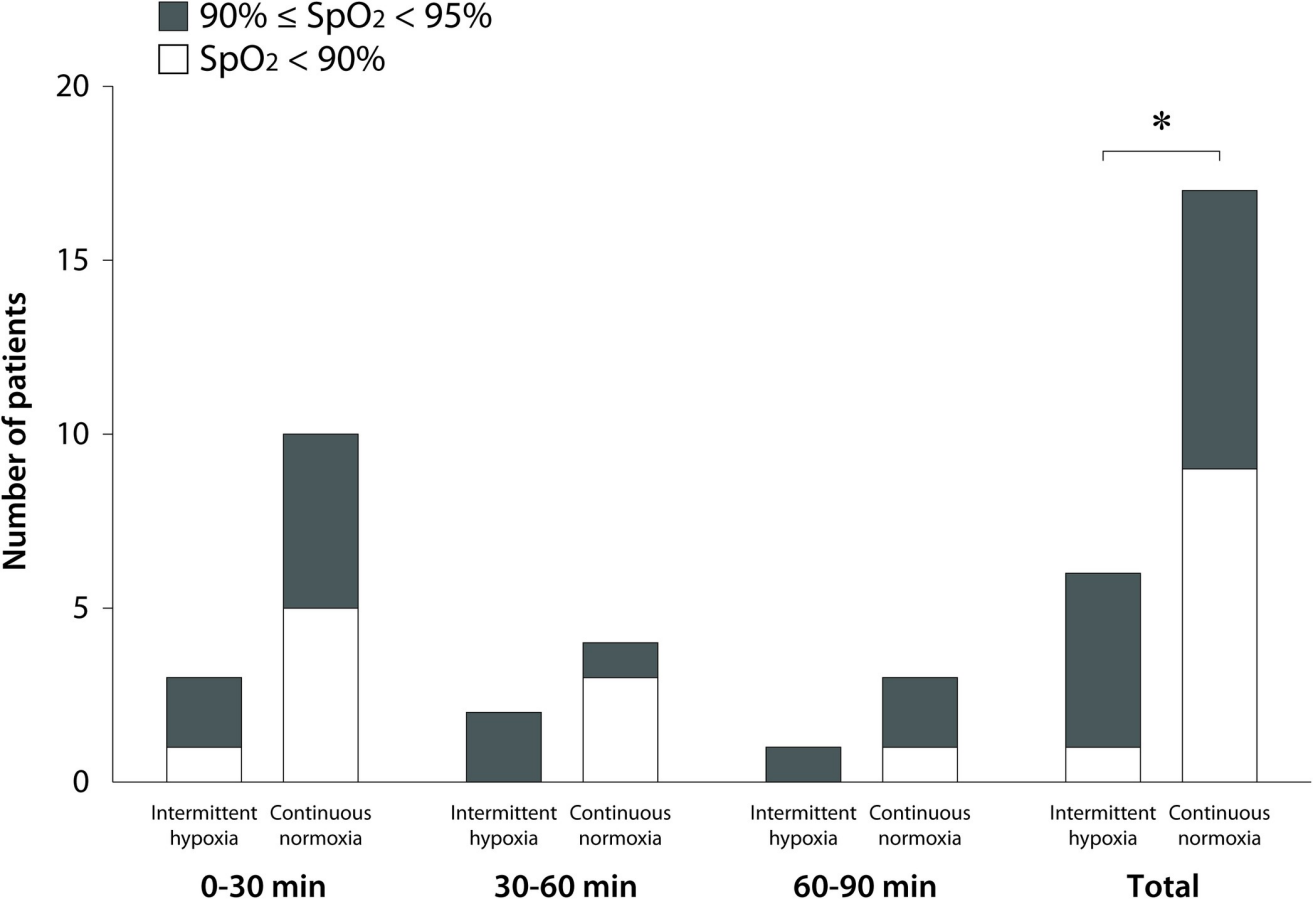
Number of hypoxemic patients with SpO_2_ <95% for 0–30, 30–60, and 60–90 min after starting one-lung ventilation. *6/68 (8.8%) vs 17/68 (25.0%), risk ratio (95% CI) 0.35 (0.15–0.84), P = 0.012 by chi-squared test for SpO_2_ <95%; 1/68 (1.5%) vs 9/68 (13.2%), risk ratio (95% CI) 0.11 (0.01–0.85), P = 0.017 by Fisher’s exact test for SpO_2_ <90%.

During the experimental protocol for 20 min before OLV, SpO_2_ was 98–100% in both groups. During OLV, the number of hypoxemic patients with SpO_2_ <95% [primary outcome; 6/68 (8.8%) vs 17/68 (25.0%), risk ratio (95% CI) 0.35 (0.15–0.84), P = 0.012 by chi-squared test] and severely hypoxemic patients with SpO_2_ <90% [1/68 (1.5%) vs 9/68 (13.2%), risk ratio (95% CI) 0.11 (0.01–0.85), P = 0.017 by Fisher’s exact test] were fewer in the intermittent hypoxia group than in the continuous normoxia group (Fig 2). Out of 23 hypoxemic patients in both groups, hypoxemia was observed within 30 min of starting OLV in 13 patients (52.5%), and within 60 min of starting OLV in 19 patients (82.6%). Hypoxemia was observed with an FiO_2_ 0.8, PEEP 5 cm H_2_O, and I:E ratio 1:2 in both groups (n = 20) except for one patient in the intermittent hypoxia group and two patients in the continuous normoxia group with an FiO_2_ 0.6, PEEP 5 cm H_2_O, and I:E ratio 1:2 at 60–90 min after starting OLV. No patient experienced more than one episode of hypoxemia.

**Fig 3:**
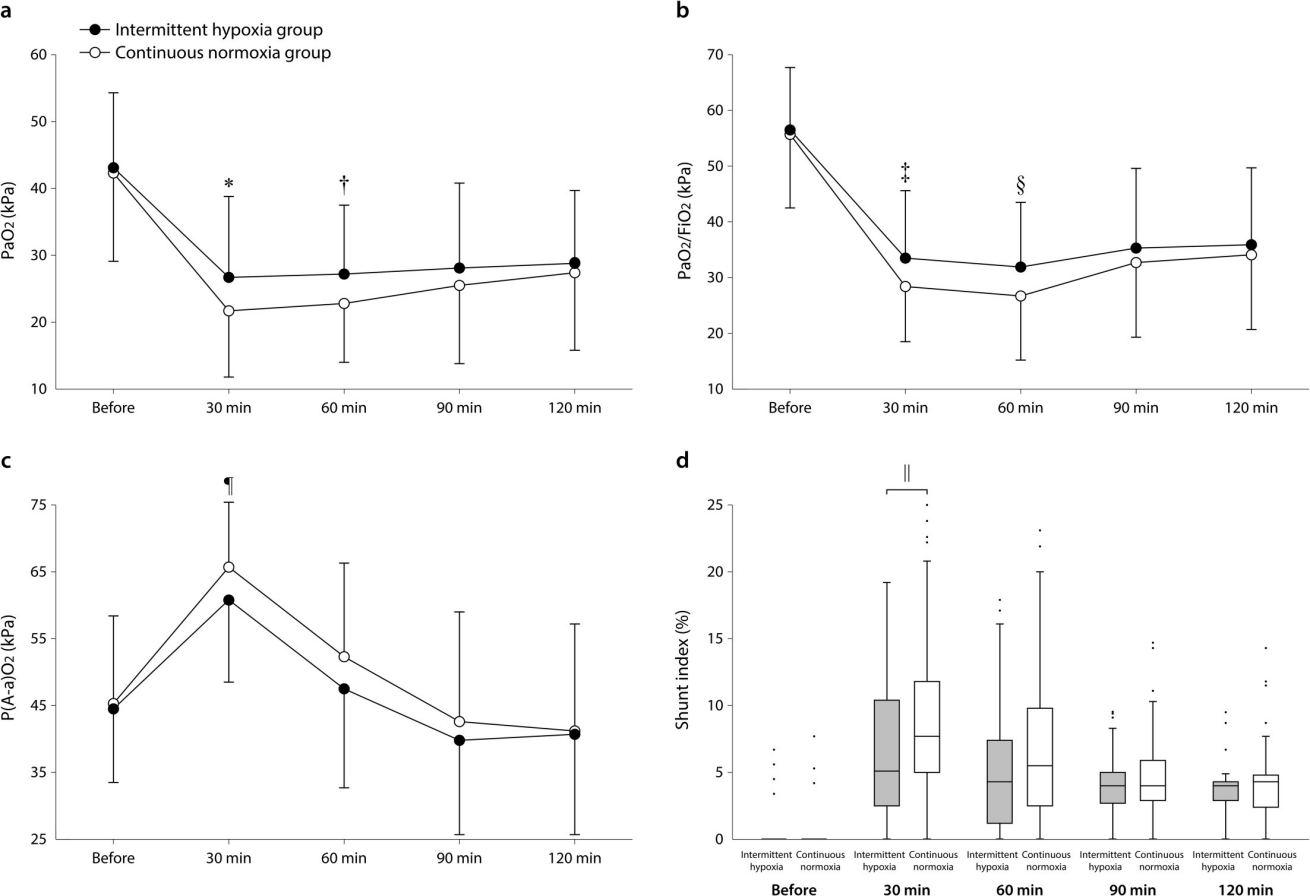
PaO_2_ (a), PaO_2_/FiO_2_ (b), alveolar-arterial oxygen pressure gradient [P(A-a)O_2_; c], and shunt index (d) before and 30, 60, 90, and 120 min after starting one-lung ventilation. The circles and error bars are mean and standard deviation. *mean difference (95% CI) 5.0 (1.3–8.8) kPa, P = 0.008; ^†^4.4 (1.2–7.6) kPa, P = 0.007; ^‡^5.0 (1.3–8.8) kPa, P = 0.008; ^§^5.3 (1.3–9.2) kPa, P = 0.008; ^¶^4.9 (-8.8 to -1.2) kPa, P = 0.010; ^║^median (interquartile range) 5.1% (2.5–10.4%) vs 7.7% (5.0–11.8%), P = 0.008.

The interactions between time and group were significant in the PaO_2_, PaO_2_/FiO_2_, P(A-a)O_2_, and shunt index (P<0.001). In within-group comparisons, compared with the baseline, the PaO_2_ and PaO_2_/FiO_2_ were lower, and the shunt index was higher 30, 60, 90, and 120 min after starting OLV; and P(A-a)O_2_ was greater 30 and 60 min after starting OLV (Fig 3; all P<0.001). In between-group comparisons, the PaO_2_ and PaO_2_/FiO_2_ were higher 30 and 60 min after starting OLV, and the P(A-a)O_2_ and shunt index were lower 30 min after starting OLV in the intermittent hypoxia group than in the continuous normoxia group (Fig 3). There were no significant interactions between time and group and no significant differences between groups during OLV in the minute ventilation (P = 0.263 by linear mixed effects model), alveolar dead space (P = 0.845), static (P = 0.723) and dynamic (P = 0.800) pulmonary compliances, FiO_2_ (P = 0.991), PEEP (P = 0.993), I:E ratio (P = 0.997), PaCO_2_ (P = 0.926), hematocrit (P = 0.785), mean arterial pressure (P = 0.688), heart rate (P = 0.445), and cardiac index (P = 0.332).

Postoperative adverse events did not differ significantly between groups (Table 2). No patient received intraoperative transfusion and postoperative mechanical ventilation.

**Table 2:** Postoperative outcomes.

	Intermittent hypoxia group (n = 68)	Continuous normoxia group (n = 68)	P-value
PaO_2_ (kPa)*	24.1 (9.6)	25.2 (8.5)	0.502
PaCO_2_ (kPa)*	6.7 (0.9)	6.7 (0.8)	0.930
Hematocrit (%)	39.1 (4.4)	39.0 (4.5)	0.875
Duration of chest tube drainage (day)	3.7 (2.3)	4.0 (3.7)	0.620
Length of stay			
Post-anesthesia care unit (min)	45.0 (16.1) (n = 49)	44.1 (14.1) (n = 51)	0.751
Intensive care unit (h)	22.5 (7.9) (n = 19)	25.9 (13.2) (n = 17)	0.347
Hospital (day)	5.0 (2.9)	5.3 (3.7)	0.610
Adverse events			0.507
Atelectasis	1	1	
Pneumothorax	1	1	
Pleural effusion	1	4	
Prolonged air leak	1	1	
Atrial fibrillation	0	2	
Chylothorax	0	1	

Data are mean (standard deviation) or number of patients. Unpaired t-tests were used to compare PaO_2_, PaCO_2_, Hematocrit, duration of chest tube drainage, and length of stay; and a Fisher’s exact test was used to compare adverse events between groups.

*Measured while O_2_ was supplied via a facemask at a rate of 5 liter min^-1^.

## Discussion

In previous human [1] and animal studies [32, 33], HPV seemed to be maximized 30–60 min after the onset of hypoxia. Thus, the risk of hypoxemia may be high until HPV has been maximized and the intrapulmonary shunt has been minimized after starting OLV [7, 10, 12]. In both groups of our study, 82.6% of hypoxemic patients with SpO_2_ <95% were found within 60 min of starting OLV. However, the incidence of hypoxemia was lower during OLV, and the PaO_2_/FiO_2_ were higher 30 and 60 min after starting OLV in the intermittent hypoxia group than in the continuous normoxia group.

During OLV, hypoxemia can develop not only by the intrapulmonary shunt in the non-ventilated lung but also by the ventilation-perfusion mismatch in the ventilated lung [9, 21] or hemodynamic instability [4]. In our study, no differences were found between groups in the respiratory or hemodynamic variables during OLV. However, the P(A-a)O_2_ and shunt index were lower 30 min after starting OLV in the intermittent hypoxia group than in the continuous normoxia group. Because the two parameters are known to be positively associated with the amount of right-to-left shunt [9, 29], these findings may suggest that the intrapulmonary shunt was smaller in the intermittent hypoxia group than in the continuous normoxia group 30 min after starting OLV.

In order to evaluate HPV and to calculate the shunt fraction, a pulmonary artery catheter is needed to measure the pulmonary artery pressure, mixed venous oxygen content, and cardiac output [4, 5, 11, 14, 28]. However, we did not use the catheter because of its invasiveness [34]. Instead of the shunt fraction requiring the mixed venous oxygen content [9, 27, 28], we calculated the shunt index using ScvO_2_ measured in the superior vena cava. The trend over time is known to be similar in the shunt fraction and the shunt index under comparable FiO_2_, PaCO_2_, and hematocrit levels, although their absolute values differ [29]. In addition, the arterial waveform analysis can estimate the cardiac output reliably in stable hemodynamic status with regular cardiac rhythms as in our study [35]. In our study, no differences were found between groups in the FiO_2_, PEEP, I:E ratio, PaCO_2_, hematocrit, and cardiac index during OLV. Therefore, the fewer hypoxemic patients and higher PaO_2_/FiO_2_ might be due to a smaller amount of the intrapulmonary shunt and greater HPV response in the intermittent hypoxia group than in the continuous normoxia group.

Previous human [36] and animal [5] studies reported that the HPV response was initiated within 2 min of the onset of hypoxia. However, the offset of HPV seems to take longer when normoxia is restored [7, 8, 37]. Therefore, if hypoxic and normoxic stimuli are applied for the same duration, the pulmonary arteries may be in a more constricted state than the baseline state, so the subsequent vasoconstriction can be augmented by the next hypoxic stimulus [7, 8]. However, previous animal studies reported that HPV was no longer augmented after 4–5 cycles of hypoxic and normoxic stimuli [14, 15]. Based on these findings, we designed the experimental protocol that the operative lung was not ventilated for 2 min and then ventilated for 2 min five times. Because this protocol only takes 20 min, we were able to apply it during surgical preparation without delaying the operation.

The effects of repeated intermittent hypoxic stimuli on HPV was studied in various experimental settings and showed inconsistent findings. Several animal studies reported that repeated hypoxic stimuli potentiated HPV in two lungs [15], one lung [13], and one lobe [12, 14]. On the other hand, in another human [6, 27] and animal studies [11], HPV was maximized by the first hypoxic stimulus for 30–60 min and was not potentiated by the subsequent hypoxic stimuli. However, out of 23 hypoxemic patients in our study, 13 (56.5%) were found within 30 min of starting OLV, whereas no patient showed hypoxemia during the experimental protocol for 20 min. Therefore, although we compared five intermittent hypoxic stimuli only with one continuous normoxic stimulus but not with one continuous hypoxic stimulus, it may be more reliable to apply multiple short-term hypoxic stimuli than one long-term hypoxic stimulus for patient safety. However, our study does not suggest the most effective and safest protocol to prevent hypoxemia during OLV.

Hypoxemia is generally defined as a PaO_2_ less than 8.0–10.7 kPa (60–80 mmHg) [10, 19, 21]. However, PaO_2_ is not continuously monitored although hypoxemia should be immediately managed during OLV. Based on previous studies [21, 24–26], we thus defined hypoxemia as a SpO_2_ <95% and severe hypoxemia as a SpO_2_ <90%, which are approximated with a PaO_2_ <10.0 kPa (75 mm Hg) and <8.0 kPa (60 mm Hg) in the oxyhemoglobin dissociation curve, respectively [10, 21, 26].

Before OLV, the alveolar recruitment strategy is another method to prevent intraoperative hypoxemia by reducing atelectasis and alveolar dead space [38]. This strategy can be used both for thoracic and non-thoracic surgery, but the high airway pressure may lead to ventilator-induced lung injury and hemodynamic instability [39]. Our intermittent hypoxic stimulating method is unlikely to cause these complications, although our method can be applied only before thoracic surgery with OLV.

Our study has limitations. We changed the FiO_2_, PEEP, and I:E ratio according to a PaO_2_ and SpO_2_ in order to provide adequate oxygenation during OLV. This was likely to confound the observed treatment effect although these parameters did not differ significantly between groups. In addition, patients with lower cardiopulmonary reserve are more vulnerable to intraoperative hypoxemia [10], but our patients were relatively healthy. We also only studied thoracic surgical patients receiving intravenous anesthesia with propofol and remifentanil in the lateral decubitus position. Because the HPV response can be affected by the type of anesthetic drugs or patient position [3, 7, 8], our findings cannot be extrapolated to patients with poor cardiopulmonary functions or with inhalational anesthesia in the supine position. Furthermore, as well as intraoperative hypoxemia, postoperative lung injury is a critical complication associated with thoracic surgery [10, 40]. However, we did not investigate it although previous studies [17, 18] suggested that repeated intermittent hypoxic stimuli would reduce lung injury by the similar preventive mechanism of remote ischemic preconditioning [41, 42]. In addition, for alveolar hypoxia, the operative lung was passively deflated but was not actively ventilated with inert gases such as nitrogen [6, 12–15, 27], in order to make a clinically applicable protocol. Out study also has potential sources of attrition and reporting biases because of missing data in the several secondary outcomes and missing outcomes in the clinical trial registry (NCT03282032), and this may decrease the validity of our study.

## Conclusions

In conclusion, repeated intermittent hypoxic stimuli to the operative lung seemed to potentiate HPV. As a result, it improved oxygenation and reduced hypoxemia during the subsequent OLV for thoracoscopic surgery in the lateral decubitus position.

## Supporting information

S1 Checklist CONSORT checklist. (DOC)

S1 Protocol Study protocol (Korean). (DOCX)

S2 Protocol Study protocol (English). (DOCX)

S1 Dataset Dataset of the study. (XLSX)

S1 Fig Experimental protocol before one-lung ventilation. (TIF)
