# Supplementary material for: Repeated intermittent hypoxic stimuli to operative lung reduce hypoxemia during subsequent one-lung ventilation for thoracoscopic surgery: A randomized controlled trial
Source: PLoS One. 2021 Apr 15;16(4):e0249880. doi: 10.1371/journal.pone.0249880 (PMC8049270; doi:10.1371/journal.pone.0249880)
Supplement: S1 Protocol — (DOCX) [file pone.0249880.s002.docx]

**일측폐환기 전조건화가 폐보호에 미치는 영향**

**Preconditioning of one-lung ventilation for lung protection**

**Version No: 1.1**

**책임연구자 소속: 마취통증의학과**

**책임연구자 이름: 서정화**

**연구 개요**

| 연구제목 | (국문) 일측폐환기 전조건화가 폐보호에 미치는 영향 |
| --- | --- |
|  | (영문) Preconditioning of one-lung ventilation for lung protection |
| 책임연구자 | 마취통증의학과 서정화 부교수 |
| 연구비 지원기관 | 없음 |

| 연구 목적 | 흉부수술 시 시야 확보를 위해 일측폐환기(one-lung ventilation)가 필요하다.  하지만 이는 수술 중 저산소증과 수술 후 폐손상 등의 합병증을 유발할 수 있다. 일부 동물 실험에서 허혈성 전조건화(ischemic preconditioning)가 이런 합병증을 줄일 수 있다는 가능성이 제기되었으나 임상연구는 아직 없다. 그러므로 폐 수술을 받는 환자에서 preconditioning이 one-lung ventilation으로 인한 합병증을 감소시키는지 알아보고자 한다. |
| --- | --- |
| 연구 설계 | 전향적 무작위 임상 연구 |
| 연구 기간 | IRB승인일 - 1년 |
| 연구 대상  (시험약 등) | 측와위 자세로 one-lung ventilation 하에 정규 폐수술을 받는 20-70세 환자 |
| 연구 대상자 수 | 각 군당 74명, 총 148명 |
| 취약한 연구대상자 | 없음 |
| 연구 방법 | 측와위 자세에서 일측폐환기 하에 정규 폐수술을 받는 성인 환자에서 수술 전에 “one-lung ventilation 2분 후 two-lung ventilation 2분"을 5 회 시행하여 preconditioning을 시행한 군(시험군)과 수술 시작까지 계속 two-lung ventilation을 유지한 군(대조군)으로 무작위 배정한다. 이후 양군 모두 수술중 일측폐환기 하에 수술을 진행한다. 수술 중 및 수술 후 폐산소화 지표(PaO_2_/F_I_O_2_, central venous oxygen saturation, incidence of hypoxemia), 폐합병증 여부를 비교함으로써 preconditioning의 폐보호 효과를 알아본다. |
| 유효성 평가 | 이 연구의 일차 임상 종점은 일측폐환기 30분 후 PaO_2_ / F_I_O_2_ ratio 이다. |
| 안전성 평가 | 본 시험에 참여한 모든 환자를 대상으로 안전성 평가를 실시한다. |
| 기대효과 및  예상결과 | 일측폐환기 preconditioning을 시행할 경우 폐보호 효과가 있을 것으로 기대한다. 이 연구를 통해 preconditioning이 임상적 이득이 있다고 밝혀지면 폐수술을 받는 환자에서 수술 전에, 수술 시간의 지체 없이 쉽고 간편하게 이 intervention을 적용하여 one-lung ventilation으로 인한 합병증을 감소시키는 효과를 기대할 수 있다. |

**연구계획서**

1. **연구 제목**

일측폐환기 전조건화가 폐보호에 미치는 영향

Preconditioning of one-lung ventilation for lung protection

1. **연구 실시기관 명칭 및 주소**

서울대학교병원 마취통증의학과, 서울특별시 종로구 대학로 101

1. **연구책임자 및 공동연구자 성명 및 직명**
2. **연구책임자**

서정화

부교수

1. **연구담당자**

윤수지

전임의

1. **임상시험용 의약품 관리약사 / 임상시험용 의료기기 관리자**

해당 없음

1. **연구 의뢰기관
   1) 연구 의뢰기관 명칭 및 주소**

해당 없음 **2) 모니터요원 성명 및 직명**

허민

진료교수

1. **연구비 지원기관 명칭 및 주소**

해당 없음

1. **예상연구기간**

IRB 승인일 – 1년

1. **연구 대상 질환**

One-lung ventilation 하에 수술이 필요한 폐질환

1. **연구의 배경 및 목적**
2. **연구 배경**

폐, 식도, 흉선 등 흉곽 내 장기의 수술 시야 확보를 위해 one-lung ventilation이 필요하다. 하지만 이는 non-ventilated lung에 많은 양의 shunt blood flow를 초래한다. 또한 one-lung ventilation에서 two-lung ventilation으로 전환 시 oxygen free radical의 생성이 증가하게 된다. 이로 인해 수술 중 저산소증과 수술 후 폐 손상 등의 합병증이 생길 수 있다.(1, 2)

짧은 ischemia 자극을 반복적으로 organ에 주면 점차 그 자극에 적응이 되어 이후 장시간의 ischemic injury를 가해도 조직 손상이 적어질 수 있다.(3) 이러한 ischemic preconditioning은 심장, 간, 신장 등에서 organ protection 효과가 있음이 여러 연구들에서 밝혀졌다.(4-7) 또한 이 preconditioning이 one-lung ventilation 동안 non-ventilated lung에서 hypoxic pulmonary vasoconstriction를 강화하여 shunt blood flow를 줄이고 oxygenation을 향상시킨다고 일부 동물 실험에서 보고하였다.(8-10) 하지만 이런 physiology가 사람에서도 보이는지, 더 나아가 one-lung ventilation으로 인한 합병증을 감소시키는 지에 대해서 성인을 대상으로 연구된 바가 없다.

**2) 연구 가설 및 목적**
그러므로 이 임상연구를 통해 ischemic preconditioning이 one-lung ventilation 하에 폐수술을 받는 환자에서 수술 중 저산소증과 수술 후 폐손상을 감소시키는 효과가 있는지 보고자 한다.

1. **시험용 의약품의 코드명이나 주성분의 일반명, 원료약품 및 그 분량, 제형 등**

해당 없음

**시험용 의료기기(분류번호/등급)의 개요 (사용 목적, 대상질환 또는 적응증을 포함)**

해당 없음

**시험용 중재 대상(수술법, 진단법 등)**

해당 없음

1. **연구대상자의 선정 기준, 제외 기준, 목표한 대상자 수 및 산출 근거**
2. **선정기준**

측와위 자세에서 one-lung ventilation 하에 정규 폐수술을 받는 20-70세 환자

1. **제외기준**

연구 참여를 거부한 환자

극심한 심혈관계 질환, 만성 폐쇄성 폐질환을 가진 환자

이전 폐수술 기왕력을 가진 환자

Pneumonectomy가 예정된 환자

1. **목표한 대상자 수 및 산출 근거**

이 연구의 일차 임상 종점은 수술 중 one-lung ventilation 시작 30분 후 PaO_2_/F_I_O_2_ ratio(P/F ratio)이다. 본원에서 폐 수술을 받은 환자 30명의 마취 기록을 검토해본 결과 one-lung ventilation 동안 P/F ratio 의 평균은 170.8, 표준편차 70.5 이었다. P/F ratio 값의 20% 차이를 임상적으로 유의하다고 보고 type I error risk 0.05, power 80% 통계분석에서 두 군간의 유의한 차이를 도출하기 위해 필요한 최소 대상자 수는 군당 67명이었다. 이에 탈락률 10%를 가정하여 군당 74명, 총 148명을 연구대상자 모집을 목표로 하였다.

1. **연구 대상자 모집 계획**

측와위 자세에서 one-lung ventilation 하에 정규 폐수술을 시행받기로 예정된 20-70세 환자들 중 별첨된 설명문과 동의서에 따라 연구 담당자가 연구 계획 및 의의에 대해 충분히 설명한 후, 서면으로 작성된 동의서에 응한 환자를 대상으로 한다. 본 연구의 책임 연구자 및 의료기관은 인종이나 사회 경제적 상태에만 근거하여 이 연구에 참여할 가능성이 있는 환자를 배제시키지 않을 것이다.

1. **연구 방법**
2. **구체적인 연구방법**
3. 마취 유도

Propofol, remifentanil을 effect-site target-controlled infusion을 통해 정맥 내로 투여하여 환자 의식을 소실시킨 후 Rocuronium을 투여하여 근이완을 유도한다. One-lung ventilation을 위해 double-lumen tube (Humanbroncho; Insung Medical, Seoul, Korea)를 삽관하고 fiberoptic bronchoscopy (LF-DP; Olympus Optical Co., Tokyo, Japan) 으로 기관지 내에 정확히 거치시킨다. Blood pressure, stroke volume, cardiac output의 모니터링과 ABGA 검사를 위해 radial artery에 catheter를 거치하고 arterial waveform analysis system (FloTrac; Edwards Lifesciences, Irvine, CA, USA)에 연결한다. Central vein의 pressure와 oxygen saturation 모니터링을 위해 internal jugular vein에 oximetry catheter (PreSep; Edwards Lifesciences, Irvine, CA, USA)를 삽입한다.

(2) Preconditioning

마취 유도 후 다음과 같이 두 군으로 무작위 배정한다.

- 시험군: 수술 전 preconditioning 시행.
- 대조군: 수술 전까지 two-lung ventilation 유지.

수술 시작 전 preconditioning을 위해 "one-lung ventilation 2분 후 two-lung ventilation 2분"을 총 5회 시행한다(총 20분 소요). One-lung ventilation은 수술 반대쪽, 즉 dependent lung에서 시행한다. One-lung ventilation의 경우 F_I_O_2_ 100%, tidal volume 6 mL·kg^-1^로 시행하고 two-lung ventilations은 F_I_O_2_ 50%, tidal volume 8 mL·kg^-1^로 각각 시행한다. 두 경우 모두 PEEP 5 cmH_2_O, respiratory rate 12 min^-1^을 공통으로 적용한다. Preconditioning이 끝나면 수술 전까지 two-lung ventilation을 유지한다.

(3) One-lung ventilation

Surgical incision 직전부터 one-lung ventilation을 시작한다. F_I_O_2_는 100% 적용 후 PaO_2_가 100-200 mmHg에서 유지되도록 조절한다. Bispectral index (A-2000 XP; Aspect Medical Systems, Newton, MA, USA) 40-60의 마취 심도를 유지하도록 propofol, remifentanil의 주입 속도를 조절하며, Train-of-four count = 0 의 근이완 상태가 되도록 rocuronium을 투여한다. Mean blood pressure < 60 mmHg, urine output < 0.5 mL·kg^-1^·h^-1^, stroke volume variation > 13%일 때 수액 200 ml, 혹은 ephedrine, phenylephrine 등의 승압제를 적절히 투여하면서 활력징후를 조절한다.

(4) 수술 종료

Two-lung ventilation을 하고 환자 체위를 supine position으로 바꾼다. Propofol과 remifentanil 주입을 중단하고 sugammadex를 투여하여 근이완을 역전시킨다. Double-lumen tube를 발관하고 마스크로 O_2_ 공급하면서 회복실 혹은 중환자실로 이송한다.

1. **비교군 설정 및 무작위 배정 방법**
2. 수술 전 preconditioning 시행하는 군 (시험군)
3. 수술 전 two-lung ventilation으로 유지하는 군 (대조군)

선정 기준에 부합하고 제외 기준에 부합하지 않는 연구대상자들을 난수표에 의해 위와 같이 두 군으로 무작위 배정한다.

무작위 배정은 이 시험에 참여하지 않는 연구자가 computer generated randomization (http://www.randomization.com)을 통해 대조군과 시험군을 각각 2명씩 포함하는 크기가 4인 block과, 각각 3명씩 포함하는 크기가 6인 block이 무작위로 섞인 block 무작위 배정표를 난수표에 따라 미리 작성하고, 이 배정표에 따른 순서대로 연구대상자를 대조군이나 시험군에 배정한다. 이때 작성한 난수표를 재현하는데 필요한 seed number, 즉 일련번호를 기록하여 보관한다. 단, 무작위 배정표는 연구대상자가 시험에 등록되기 전에 연구에 참여하는 연구대상자를 볼 수 없는 연구와 독립된 제 3자에 의해 만들도록 하며 운영, 관리되어 Allocation concealment를 유지하도록 한다.

1. **시험약 투여∙사용량, 투여∙사용 방법, 병용 요법, 대조약 사용시 그 선택 사유 등**

**시험 의료기기 사용량∙사용방법∙사용기간∙병용요법 등**

**시험 중재에 대한 설명**

본 연구에서의 preconditioning은 수술 시작 전 미리 one-lung ventilation에 노출시키는 방법으로, 마취 유도 후 시험군으로 무작위 배정된 환자를 대상으로 시행한다. 수술 시작 전 preconditioning을 위해 "one-lung ventilation 2분 후 two-lung ventilation 2분"을 총 5회 double lumen tube를 사용하여 시행한다. One-lung ventilation은 수술 반대쪽, 즉 dependent lung에서 시행한다. One-lung ventilation의 경우 F_I_O_2_ 100%, tidal volume 6 mL·kg^-1^로 시행하고 two-lung ventilations은 F_I_O_2_ 50%, tidal volume 8 mL·kg^-1^로 각각 시행한다. 두 경우 모두 PEEP 5 cmH_2_O, respiratory rate 12 min^-1^을 공통으로 적용한다. Preconditioning이 끝나면 수술 전까지 two-lung ventilation을 유지한다.

1. **관찰항목, 임상검사항목 및 관찰검사방법**
2. 일차 임상 종점

: one-lung ventilation 시작 30분 후 PaO_2_/F_I_O_2_ ratio (P/F ratio)

1. 이차 임상 종점

**Oxygenation**

- 60, 90, 120분 후 P/F ratio

- One-lung ventilation 중 가장 낮게 측정된 P/F ratio

- Central venous oxygen saturation (ScvO_2_)

- Incidence of hypoxemia (SpO_2_ < 95%)

**Postoperative outcomes**

- Pulmonary complications: Acute lung injury, acute respiratory distress syndrome, atelectasis, pneumonia 등

- Pulmonary function tests (FVC, FEV_1,_ FEV_1_/FVC, DLCO)

- Length of stay in intensive care unit or hospital

**Intraoperative cardiopulmonary outcomes**

- Blood pressure, heart rate

- Stroke volume (SVV), cardiac output (CO)

- Central venous pressure

- Static and dynamic pulmonary compliances

- PaCO_2_

- Amounts of inotropics, fluid, and transfusion

- Any perioperative adverse events

(3) 관찰항목, 임상검사 항목

① 수술 전

- 성별, 나이, 체중, 키, 기저질환, 진단명, 수술명, 수술 전 폐기능 검사(FVC, FEV_1_, FEV_1_/FVC, DLCO), 동맥혈 가스분석, Chest radiograph.

② 수술 중

- 동맥혈 가스분석, 저산소증 빈도, hemodynamic data(CO, SVV, ScvO_2_), ventilatory data, 수술 중 약물 주입량, 수혈량, 수액주입량, 소변량, 추정 실혈량, 총 일측폐환기 시간, 총 수술 시간, 총 마취 시간.

③ 수술 후

- 동맥혈 가스분석, 폐기능 검사(FVC, FEV_1_, FEV_1_/FVC, DLCO), Chest radiograph, acute lung injury, adult respiratory distress syndrome 등의 lung complications, 중환자실 및 병원 총 체류시간.
- 수술 후 평가항목인 동맥혈 가스분석, 폐기능검사, chest radiograph의 경우 수술 직후 중환자실에서 평가하며, lung complications, 중환자실 및 병원 총 체류시간은 이후 병록 기록의 review를 통해 시행한다.

(4) 관찰검사 방법

① 동맥혈 가스분석(Arterial blood gas analysis, ABGA)

- PaO_2_, SaO_2_, PaCO_2_, pH를 다음과 같은 시점에 측정한다. 이때 F_I_O_2_와 E_T_CO_2_를 함께 기록한다.

i) 마취 유도 후 앙와위 자세에서 Preconditioning 직전

ii) 수술 시작 후 one-lung ventilation 30, 60, 90, 120분 후

iii) 수술 종료 직후

② Hypoxic event

- 수술 중 one-lung ventilation 동안 말초혈액 산소포화도(SpO_2_)가 95% 미만으로 감소한 환자의 수를 기록한다.

③ Ventilatory data

- ABGA 측정과 동일한 시점에 호기말 이산화탄소 농도(E_T_CO_2_), peak inspiratory pressure(Ppeak), mean inspiratory pressure(Pmean), plateau inspiratory pressure (Pplateau), tidal volume(Vt), respiratory rate(RR)을 기록한다.

④ Hemodynamic data

- 마취 중 연속적으로 Arterial blood pressure(systolic, diastolic, mean), heart rate, SpO_2_ 자료를 수집한다.

- 요골동맥에 연결한 FloTrac™을 통해 CO, SVV 자료를 수집한다.

- 우측 내경 정맥에 거치한 PreSep™ catheter를 통해 ScvO_2_ 자료를 수집한다.

1. **기존 치료 및 연구와의 차별점**

현재 폐수술 환자의 경우 일측폐환기시 저산소증을 예방하기 위해 수술 전에 시행하는 별도의 전처치는 없는 상태이다. 수술 중 저산소증 발생시 환기폐에 호기말양압을 적용하고, 이 방법이 비효과적이면 비환기폐의 지속적 기도양압을 적용하거나 일측폐환기를 포기하고 양측폐환기를 시도하고 있다.

이 연구의 경우 기존에 없던 수술전 일측폐환기의 preconditioning 전처치를 통해 수술중 저산소증 발생 및 폐손상 감소에 얼마나 효과가 있을지 보고자 한다.

1. **연구대상자의 이익과 위험**

일반적으로 폐절제술에서 일측폐환기시 저산소증, 급성 폐손상 등의 합병증이 생길 수 있다. 본 연구에서 수술전 시행하는 간헐적인 일측폐환기의 경우 그 방법이 임상에서 시행되는 일측폐환기 방법과 다르지 않고 그 시간이 매우 짧으며 양측폐환기로 바로 전환되므로, 본 연구로 인해 추가적인 위험이 생길 여지는 적다. 또한 모든 과정은 숙련된 마취과 전문의가 곁에서 감시하며 합병증이 발생하더라도 적절한 조치를 취하게 되므로 환자에게 추가 위험의 가능성은 매우 적을 것으로 생각된다.

1. **중지 탈락 기준**

수술적 요인에 의해 수술중 일측폐환기가 30분 미만으로 시행되는 경우

수술 중 환자의 자세에 예상치 못한 변화가 요구되는 경우

연구대상자의 동의 철회

1. **부작용을 포함한 안전성의 평가기준, 평가 방법 및 보고 방법**

본 시험에 참여한 모든 환자를 대상으로 안전성 평가를 실시한다. 연구 시행 중에 이상 반응이 발생했을 경우, 연구에 사용된 시험 방법과의 인과 관계 유무와 모든 이상 반응을 기록하고 추후 중증도, 중대성, 기간, 그리고 시험 방법과의 인과관계를 평가한다. 이상반응에 대한 처치 및 결과 역시 기록한다. 이상 반응은 시험 기간 중의 계획된 검진과 검사의 소견 이외에도 비정상적인 검진이나 필요에 따른 추가적인 검사와 검진에 의해 평가하고 즉각적인 조치를 시행한다. 임상병리검사 자료에 대해서는 변수의 특성에 따라 치료 전, 후의 군내 비교 등 적절한 통계적 방법을 이용하여 분석하고, 이상반응의 빈도, 발현률, 각각의 목록, 심각한 정도 및 시험 방법과의 인과관계 등을 제시하며, 필요한 경우 그래프 형태로 보고한다.

1. 중증도

이상반응은 아래의 정의에 따라 경증, 중등증, 중증으로 구분된다.

- 경증은 일반적이고 일시적이고 일상적인 활동을 방해하지 않는다.

- 중등증은 약간의 불편함을 초래하거나 일상적인 활동을 방해한다.

- 중증은 일상적인 활동을 수행할 수 없다.

1. 인과 관계

인과 관계는 ‘관련 없을 것으로 생각됨’, ‘관련 있을 가능성 있음’, ’가능성 많음’, ’명백히 관련 있음’, 또는 ‘관련성을 확인하기 어려움’으로 구분된다.

1. **효과 평가기준, 평가 방법 및 해석방법 (통계분석방법 등)**

무작위 군배정, 표본수 계산, 통계 분석에 STATA (Special Edition 14.2; Stata Corporation, College Station, Texas, USA)를 사용한다. 통계분석에 포함시킬 분석군은 Intention-to-treat(ITT)을 통해 분석한다. 연속변수는 Shapiro-Wilk test로 정규성 검정 후 모수 혹은 비모수 분석을 시행한다. 일회 측정의 경우 unpaired t or Mann-Whitney U tests, 반복 측정의 경우 repeated measures ANOVA, linear mixed models, or Friedman test 로 분석하며 mean difference, with 95% CI 로 표현한다. 명목변수는 Fisher’s exact test를 이용하여 분석하며 risk ratio with 95% CI 로 표현한다. *P* < 0.05을 통계적으로 유의 하다고 간주하며, post-hoc analysis는 Bonferroni correction으로 교정한 P value를 사용한다.

1. **임상시험 후 연구대상자의 진료 및 치료기준**

해당 진료과의 치료 지침을 따른다.

1. **연구수행일정표**

IRB 통과 직후부터 1년

- 1개월: 임상시험 준비

- 8개월: 임상시험

- 3개월: 자료 정리 및 논문 작성

1. **자료 및 안전성 모니터링 계획 (Data and Safety Monitoring Plan)**
2. **모니터링 책임자**

***모니터링 책임자**

서정화

서울특별시 종로구 대학로 101, 서울대학교병원 마취통증의학과

02-2072-2467

***모니터링 담당자**

허민

서울특별시 종로구 연건동 28번지, 서울대학교병원 마취통증의학과

02-2072-2467

1. **자료 및 안전성 정보 모니터링 항목**

*자료(study accruals) 항목: 마취 중 측정한 P/F ratio, ScvO_2,_ Blood pressure, heart rate, SVV, CO, ventilator data, 수술 중 약물 주입량, 주입된 수액 및 혈액량, 소변량, 추정 실혈량, 총 일측폐환기 시간, 수술후 합병증 발생유무, 중환자실 및 병원 총 체류 시간

*안전성(Safety) 항목: 산소포화도 감시를 통한 저산소증 발생 여부, 침습적 혈압 감시를 통한 저혈압 발생 여부를 모니터링 한다.

1. **자료 및 안전성 모니터링 방법 및 주기**

선정 기준에 부합하고 임상연구에 동의한 환자를 대상으로 유효성 및 안전성 분석을 시행한다. 모니터링 빈도는 6개월로 하며, 환자에게 임상 연구 시행 도중 예기치 않은 사망, 손상 등의 중대한 이상 반응이나 계획서 미 준수 등이 발생하는 경우 추가 모집을 중단하고 2주 안에 IRB에 보고하여 연구 계획서 변경 혹은 연구 지속 여부를 결정하도록 한다.

본 연구의 자료는 연구 대상자의 권리와 복지를 보호하기 위하여 모든 연구대상자의 자료를 문헌으로 작성된 증례기록지 형식 혹은 암호화된 전자파일로 작성하여 정해진 저장소에 보관한다. 본 연구는 GCP guideline에 의거하여 연구와 관계가 없는 감독관이 증례 기록지에 기록된 정보의 정확성과 안전성 그리고 검증 가능성을 확인하게 된다. 연구에 참여하는 모든 환자는 설명에 근거한 동의를 문헌으로 작성한 후 연구에 참여하게 된다.

1. **이상약물반응보고, 연구 미준수, 예상하지 못한 문제의 보고**

연구자 인지일로부터 근무일 15일 이내에 IRB에 보고 한다.

1. **연구 중단 기준**

연구 대상자의 동의 철회

연구자가 연구의 진행이 환자의 이익에 위배된다고 판단하는 경우

연구가 중단된 경우 2주 안에 IRB에 보고하여 연구 계획서 변경 혹은 연구 지속 여부를 결정하도록 한다.

1. **연구대상자의 안전보호를 위한 대책**
2. **연구의 윤리성 확보를 위한 기본 방안**

2013 헬싱키 선언에 입각하여, 대상자 또는 보호자에게 연구의 목적과 연구 참여 중 일어날 수 있는 정신적, 신체적 위해를 충분히 설명한 후 대상자 (또는 보호자) 로부터 서면동의서를 받을 예정이다. 또한 ICH-GCP를 준수하고 병원윤리위원회의 윤리규정을 준수할 것이며 IRB 승인 후 연구를 수행할 것이다.

1. **연구대상자의 동의 과정**

별첨된 설명문과 동의서에 따라 연구 담당자가 환자에게 설명한 후 서면 동의를 받는다. 동의를 제공 할 자는 연구 대상자 본인과 배우자, 자녀 등의 직계 대리인으로 한정한다. 연구 설명 후 환자가 자발적으로 동의 할 때까지 충분한 시간을 제공한다. 연구 동의 과정에는 어떠한 강제성도 없을 것이며 연구 설명은 일반인이 받아들이기 쉬운 평이한 언어로 시행할 것이다. 연구 동의를 받은 후 설명문 사본 한 부를 환자가 보관하도록 제공한다.

1. **연구대상자의 보상 방안**

연구 참여 시 비용의 차이나 시험 참여에 따르는 추가적인 보상이나 금전적 혜택은 제공되지 않는다. 하지만 본 연구 시험 실시 중 연구의 직접적인 원인에 의해서 통상적인 부작용 외 예기치 못한 부작용에 대해서 병원의 임상시험 피해자 보상규약에 따른 적절한 보상을 제공한다.

1. **연구대상자의 개인정보보호 방안**

대상자, 대상자의 담당의사, 시험 참여자 이외에는 대상자의 시험 참여 여부나 치료 경과에 대해 알지 못하게 하며, 대상자의 신원을 파악할 수 있는 기록은 비밀로 보장될 것이다. 연구를 위해 수집되는 정보는 잠금장치가 있는 연구실에 비밀번호가 걸린 파일로 보관하고 연구파일에 접근할 수 있는 사람은 권한을 가진 일부 연구원으로 제한할 것이다. 수집되는 자료의 불필요한 개인식별자는 제거하고, 특히, 증례기록서에는 환자의 이름, 주민등록번호, 차트 번호 등을 기재하지 않도록 하며, 신상정보와 연결된 식별자 코드는 별도로 관리할 것이다. 환자와 관련된 사진을 제출할 때는 환자의 신원을 알 수 없도록 할 것이며 조금이라도 신원이 노출될 가능성이 있는 경우에는 이에 대한 서면 동의를 받았음을 명시할 것이다. 대상자의 검진 기록은 비밀이 유지되고 다른 곳으로 이동되지 않을 것이며, 본 연구의 진행 여부를 감독 받기 위해 감독 기관으로 보내어질 수 있다. 연구를 위해 수집되는 데이터는 연구 종료 후 3년동안 보관한다.

1. **취약한 연구대상자를 포함하는 경우 추가적인 보호조치 방안**

해당 없음

1. **인체유래물의 보관 및 폐기 방법**

해당 없음

1. **참고 문헌**

1. Park HP, Yoon MJ, Jeon YT, Kang JM, Hwang JW, Oh YS. Which predictable variables identify patients at risk of arterial hypoxemia during one-lung ventilation?: analysis of preoperative and intraoperative variables. Korean Journal of Anesthesiology. 2005;49(2):167-71.

2. Dunn PF. Physiology of the lateral decubitus position and one-lung ventilation. International anesthesiology clinics. 2000;38(1):25-53.

3. Przyklenk K, Bauer B, Ovize M, Kloner RA, Whittaker P. Regional ischemic 'preconditioning' protects remote virgin myocardium from subsequent sustained coronary occlusion. Circulation. 1993;87(3):893-9.

4. Hausenloy DJ, Yellon DM. The therapeutic potential of ischemic conditioning: an update. Nature Reviews Cardiology. 2011;8(11):619-29.

5. Kapitsinou PP, Haase VH. Molecular mechanisms of ischemic preconditioning in the kidney. American Journal of Physiology-Renal Physiology. 2015;309(10):F821-F34.

6. Wang Y, Shen J, Xiong X, Xu Y, Zhang H, Huang C, et al. Remote ischemic preconditioning protects against liver ischemia-reperfusion injury via heme oxygenase-1-induced autophagy. PloS one. 2014;9(6):e98834.

7. Zarbock A, Schmidt C, Van Aken H, Wempe C, Martens S, Zahn PK, et al. Effect of remote ischemic preconditioning on kidney injury among high-risk patients undergoing cardiac surgery: a randomized clinical trial. The Journal of the American Medical Association. 2015;313(21):2133-41.

8. Benumof JL. Intermittent hypoxia increases lobar hypoxic pulmonary vasoconstriction. Anesthesiology. 1983;58(5):399-404.

9. Miller MA, Hales CA. Stability of alveolar hypoxic vasoconstriction with intermittent hypoxia. Journal of applied physiology. 1980;49(5):846-50.

10. Pirlo AF, Benumof JL, Trousdale FR. Potentiation of lobar hypoxic pulmonary vasoconstriction by intermittent hypoxia in dogs. Anesthesiology. 1981;55(3):226-30.
