# Supplementary material for: Repeated intermittent hypoxic stimuli to operative lung reduce hypoxemia during subsequent one-lung ventilation for thoracoscopic surgery: A randomized controlled trial
Source: PLoS One. 2021 Apr 15;16(4):e0249880. doi: 10.1371/journal.pone.0249880 (PMC8049270; doi:10.1371/journal.pone.0249880)
Supplement: S2 Protocol — (DOCX) [file pone.0249880.s003.docx]

**Clinical Study Protocol**

**(Version 1.0)**

**1. Study Title**

Preconditioning of One-lung Ventilation for Lung Protection

**2. Address of Research Center**

Department of Anesthesiology and Pain Medicine, Seoul National University Hospital, Seoul National University College of Medicine, 101 Daehak-ro, Jongno-gu, Seoul 03080, Korea

**3. Principal investigators and Co-investigators**

3.1 Principal Investigators

Jeong-Hwa Seo, Professor

Department of Anesthesiology and Pain Medicine, Seoul National University Hospital, Seoul National University College of Medicine

**4. Responsibility of trial**

Investigator-initiated trial

**5. Address of sponsorship**

None

**6. Study period**

12 months after the approval of IRB

**7. Subjects of study**

Elective thoracic surgery which requires one-lung ventilation with lateral decubitus position

**8. Necessity of Study and Overview**

8.1 Background

For lung isolation and better surgical view, one-lung ventilation(OLV) is essential for thoracic surgery. However, OLV may cause serious pulmonary complications due to intraoperative hypoxia. Animal studies showed a positive effect of OLV preconditioning (Intermittent OLV before surgical intervention) on perioperative pulmonary oxygenation.

8.2 Hypothesis and objectives

Thus, we designed a study to observe the effect of OLV preconditioning on perioperative oxygenation during thoracic surgery

**9. Study materials**

Intervention (OLV preconditioning)

**10. Inclusion and exclusion criteria**

10.1 Inclusion criteria

Patients aged 20-70 years with ASA physical status I–III who receive elective thoracic surgery which requires one-lung ventilation with lateral decubitus position

10.2 Exclusion criteria

Patients with severe cardiopulmonary diseases, history of pulmonary resection, planned bilateral lung surgery, irregular cardiac rhythm, anemia, pregnancy, and body mass index >35 kg m^-2^.

10.3 Sample size calculation

In our pilot study (n=10), three patients (30%) showed hypoxemia with SpO_2_ <95% during OLV when no pretreatment had been applied before OLV. To detect a 20% difference in the number of hypoxemic patients using a chi-squared test, 62 patients were needed in each group with an α of 0.05 and power of 0.8 for two-sided analysis.

10.4 How to recruit

Potential participants who meet the inclusion and exclusion criteria (See 10.1 and 10.2) are recruited at outpatient clinics or on the preoperative visit before surgery, and written informed consents are obtained from all of the participants.

**11. Study protocols**

11.1 Interventions

Patients are monitored with non-invasive blood pressure, pulse oximetry, electrocardiography, bispectral index (A-2000 XP; Aspect Medical Systems, Newton, MA, USA), and acceleromyography (TOF-watch Sx; Organon, Dublin, Ireland). Propofol (Fresofol MCT 2%; Fresenius Kabi, Homburg, Germany) and remifentanil (Ultiva; GlaxoSmithKline, Brentford, Middlesex, UK) are administered intravenously with effect-site target-controlled infusion (Orchestra; Fresenius Kabi, Brézins, France). The initial target concentration was 3–5 μg ml^-1^ for propofol, and 3–5 ng ml^-1^ for remifentanil.

After intravenous administration of rocuronium 0.6–0.8 mg kg^-1^, train-of-four (TOF) counts are monitored at the adductor pollicis muscle every 15 s. At a TOF count=0 and bispectral index <60, a double-lumen tube (Mallinckrodt endobronchial tube; Covidien, Mansfield, MA, USA) is placed into the left mainstem bronchus by direct or video laryngoscopy. The 32, 35, 37, or 39-Fr double-lumen tubes are selected based on the left bronchial diameter measured on the preoperative chest computed tomography, or sex and height of the patient.

With fiberoptic bronchoscopy (LF-DP or LF-GP; Olympus Optical Co., Tokyo, Japan), anesthesologist positions the bronchial cuff of the double-lumen tube into the left bronchus below the carina without herniation, and the bronchial tip above the left lobar bronchi without obstruction. If the tube is malpositioned into the right bronchus, it is repositioned into the left bronchus with bronchoscopic guidance. The tracheal and bronchial cuff pressures are adjusted to <25 cm H_2_O (VBM Medizintechnik GmbH, Sulz am Neckar, Germany).

A 20-gauge catheter is inserted into the radial artery and was connected to a transducer for arterial waveform analysis (FloTrac, version 4.0; Edwards Life Sciences, Irvine, CA, USA). An 8.5-Fr central venous oximetry catheter (PreSep; Edwards Lifesciences, Irvine, CA, USA) is inserted into the right internal jugular vein, and its tip was positioned at the junction of the superior vena cava and right atrium with sonographic guidance (Vivid i; GE Healthcare, Chicago, IL, USA) [22]. The PreSep catheter is calibrated in vivo with hemoglobin, hematocrit, and oxygen saturation (ScvO_2_) values measured in the central venous blood. The arterial and venous pressure transducers are placed at the right atrium level and were exposed to atmospheric pressure for zeroing.

After moving the patient to the lateral decubitus position, anesthesiologist adjusts the positions of the double-lumen tube and pressure transducers. In the intermittent hypoxia group, the nondependent lung is deflated for 2 min and then ventilated for 2 min while the dependent lung is continuously ventilated (Fig 1). The deflation and ventilation of the nondependent lung is repeated five times. In the continuous normoxia group, both lungs are ventilated for 20 min. In both groups, the anesthetic machine (Primus; Dräger, Lübeck, Germany) is set to an FiO_2_ 1.0 and tidal volume 6 ml kg^-1^ of predicted body weight (PBW) during deflation of the dependent lung, and to an FiO_2_ 0.5 and tidal volume 8 ml kg^-1^ of PBW during ventilation of both lungs. The PBW is calculated as 50.0+0.905×(height‑152.4) for men, and 45.5+0.905×(height‑152.4) for women [23]. A respiratory rate 12 breaths min^-1^, PEEP 5 cm H_2_O, and inspiratory:expiratory (I:E) ratio 1:2 are applied during the experimental protocol in both groups. This experimental protocol is conducted during surgical preparation by attending anesthetists, who are not the investigators of this study. Investigators did not enter the operating room during the experimental protocol for blinding of group assignment.

After the protocol, OLV is initiated with an FiO_2_ 0.8, PEEP 5 cm H_2_O, I:E ratio 1:2, tidal volume 4–8 ml kg^-1^ of PBW, and respiratory rate 12–20 breaths min^-1^ to maintain an SpO_2_ ≥95%, PaO_2_ 13.3–33.3 kPa (100–250 mm Hg), PaCO_2_ 4.7–6.7 kPa (35–50 mm Hg), and peak inspiratory pressure <30 cm H_2_O. When a PaO_2_ is ≥33.3 kPa (250 mm Hg), the FiO_2_ is decreased to 0.6; and when a PaO_2_ is <13.3 kPa (100 mm Hg), the FiO_2_ and PEEP are increased to 1.0 and 8 mm Hg, respectively. If SpO_2_ decreased to <95%, anesthesiologist applies alveolar recruitment maneuvers to the dependent ventilated lung with an FiO_2_ 1.0, end-inspiratory pressure 30 cm H_2_O, PEEP 10 cm H_2_O, and I:E ratio 1:1 until SpO_2_ increases to ≥98% after checking the double-lumen tube position with fiberoptic bronchoscopy. If SpO_2_ drops to <90%, anesthesiologist asks the surgeon to stop the operation, and ventilates both lungs manually with an FiO_2_ 1.0 until SpO_2_ increases to ≥98%. The effect-site concentrations of propofol and remifentanil are titrated for a bispectral index 30–60, and rocuronium 0.2–0.3 mg kg^-1^ is intermittently administered for a TOF count=0. Ephedrine 5–10 mg, phenylephrine 30–50 μg, or plasmalyte 100–200 ml are administered intravenously to manage a mean blood pressure <60 mm Hg, central venous pressure <4 mm Hg, stroke volume variation >13%, or urine output <0.5 ml kg^-1^ h^-1^ according to the attending anesthesiologist’s discretion, when one or more conditions are met. A packed red blood cell is transfused to maintain a hematocrit level >20%.

After pulmonary resection, the nondependent operative lung is inflated and then bilateral lungs are ventilated with an FiO_2_ 0.5. A chest tube is inserted into the operative hemithorax through the incision for the thoracoscopic port. Intravenous patient-controlled analgesia (Automed 3200; Ace Medical, Seoul, Korea) is started with a 100-ml mixture of fentanyl 1000–2000 μg, morphine 40–80 mg, ramosetron 0.3–0.6 mg, and saline at an infusion of 1 ml h^-1^, bolus of 0.5 ml, and lockout time of 10 min.

After surgery, patients are turned to the supine position and secretions are suctioned from both lungs. The double-lumen tube is removed after administration of sugammadex 4 mg kg^-1^ when the patient had spontaneous breathing, responses to verbal commands, and a TOF ratio >0.9. If mechanical ventilation is required after surgery, the double-lumen tube is replaced with a plain tube without the administration of sugammadex. Patients are transferred to post-anesthesia or intensive care units.

11.2 Randomization and Blinding

Patients are randomized into two groups. Before one-lung ventilation, in the intermittent hypoxia group, the nondependent lung is deflated for 2 min and then ventilated for 2 min while the dependent lung is continuously ventilated. This is repeated five times. In the continuous normoxia group, both lungs are ventilated for 20 min. Group assignment is randomized in a 1:1 ratio by a clinician not involved in the study Patients and investigators are blinded to group assignment.

11.3 Administration and dose of drug

N/A

11.4 Outcome measurements

- Baseline data: sex, age, body weight, height, body mass index, ASA physical status, diagnosis, comorbidity, smoker, pulmonary function test, type of surgery, side of surgery, intraoperative fluid and drug administration, duration of surgery and anesthesia

- SpO_2_ during OLV

- PaO_2_, PaCO_2_, SaO_2_, and hematocrit: Immediately before and 30, 60, 90, and 120 min after starting OLV

- FiO_2_, PEEP, I:E ratio, EtCO_2_, tidal volume, respiratory rate, and peak or plateau airway pressures

- Mean arterial pressure, heart rate, and cardiac index with the FloTrac sensor; and ScvO_2_ with the PreSep catheter

- Primary outcome: The number of patients with SpO2 <95% during the entire period of OLV.

- Secondary outcomes: The number of episodes and the number of patients with mild or severe hypoxemia during OLV; PaO_2_, PaO_2_/FiO_2_, P(A‑a)O_2_, shunt index, and respiratory or hemodynamic variables including minute ventilation, alveolar dead space, static and dynamic pulmonary compliances, FiO_2_, PEEP, I:E ratio, PaCO_2_, hematocrit, mean arterial pressure, heart rate, and cardiac index immediately before (baseline) and 30, 60, 90, and 120 min after starting OLV; duration of the chest tube drainage and lengths of stay in the postoperative care units and hospital after surgery; and perioperative adverse events.

11.6 Management of adverse events

All interventions are monitored by trained anesthesiologists and if complications occur, appropriate treatments are provided to decrease additional risk to the patient.

11.7 Criteria for Discontinuation and Drop-out

Withdrawal of consent

11.8 Plan for report of harmful cases

Reports of serious adverse events (hypoxemia problems such as hypoxic brain damage, hypoxic organ damage, etc.) and unexpected problems and research-related adverse events are reported to the IRB within 7 days, and within 24 hours for serious and unexpected adverse events.

11.9 Data Safety Monitoring Plan

Principal investigator will make comparisons between the source documents and the study protocol at every 6 months to assure the completeness of data and will review the safety data of subjects. The subject identification code on the data will be encoded, and will be filed in a safe installed with a locking device while the electronic document will be saved in a computer which is restricted for access.

**12. Statistical analysis**

Continuous variables are summarised as mean (standard deviation) or median (interquartile range) and analyzed with unpaired or paired t-tests and Mann-Whitney U or Wilcoxon signed-rank tests. Repeatedly measured longitudinal data are analyzed with linear mixed effects models. Categorical variables are presented as the number of patients (%) and compared with Fisher’s exact test. Effect sizes with 95% confidence intervals are calculated. All the statistical analyses were two-sided and a significance criterion was P <0.05.

**13. References**

1. Park HP, Yoon MJ, Jeon YT, Kang JM, Hwang JW, Oh YS. Which predictable variables identify patients at risk of arterial hypoxemia during one-lung ventilation?: analysis of preoperative and intraoperative variables. Korean Journal of Anesthesiology. 2005;49(2):167-71.

2. Dunn PF. Physiology of the lateral decubitus position and one-lung ventilation. International anesthesiology clinics. 2000;38(1):25-53.

3. Przyklenk K, Bauer B, Ovize M, Kloner RA, Whittaker P. Regional ischemic 'preconditioning' protects remote virgin myocardium from subsequent sustained coronary occlusion. Circulation. 1993;87(3):893-9.

4. Hausenloy DJ, Yellon DM. The therapeutic potential of ischemic conditioning: an update. Nature Reviews Cardiology. 2011;8(11):619-29.

5. Kapitsinou PP, Haase VH. Molecular mechanisms of ischemic preconditioning in the kidney. American Journal of Physiology-Renal Physiology. 2015;309(10):F821-F34.

6. Wang Y, Shen J, Xiong X, Xu Y, Zhang H, Huang C, et al. Remote ischemic preconditioning protects against liver ischemia-reperfusion injury via heme oxygenase-1-induced autophagy. PloS one. 2014;9(6):e98834.

7. Zarbock A, Schmidt C, Van Aken H, Wempe C, Martens S, Zahn PK, et al. Effect of remote ischemic preconditioning on kidney injury among high-risk patients undergoing cardiac surgery: a randomized clinical trial. The Journal of the American Medical Association. 2015;313(21):2133-41.

8. Benumof JL. Intermittent hypoxia increases lobar hypoxic pulmonary vasoconstriction. Anesthesiology. 1983;58(5):399-404.

9. Miller MA, Hales CA. Stability of alveolar hypoxic vasoconstriction with intermittent hypoxia. Journal of applied physiology. 1980;49(5):846-50.

10. Pirlo AF, Benumof JL, Trousdale FR. Potentiation of lobar hypoxic pulmonary vasoconstriction by intermittent hypoxia in dogs. Anesthesiology. 1981;55(3):226-30.
