# Supplementary figures and images for: Repeated intermittent hypoxic stimuli to operative lung reduce hypoxemia during subsequent one-lung ventilation for thoracoscopic surgery: A randomized controlled trial
Source: PLoS One. 2021 Apr 15;16(4):e0249880. doi: 10.1371/journal.pone.0249880 (PMC8049270; doi:10.1371/journal.pone.0249880)

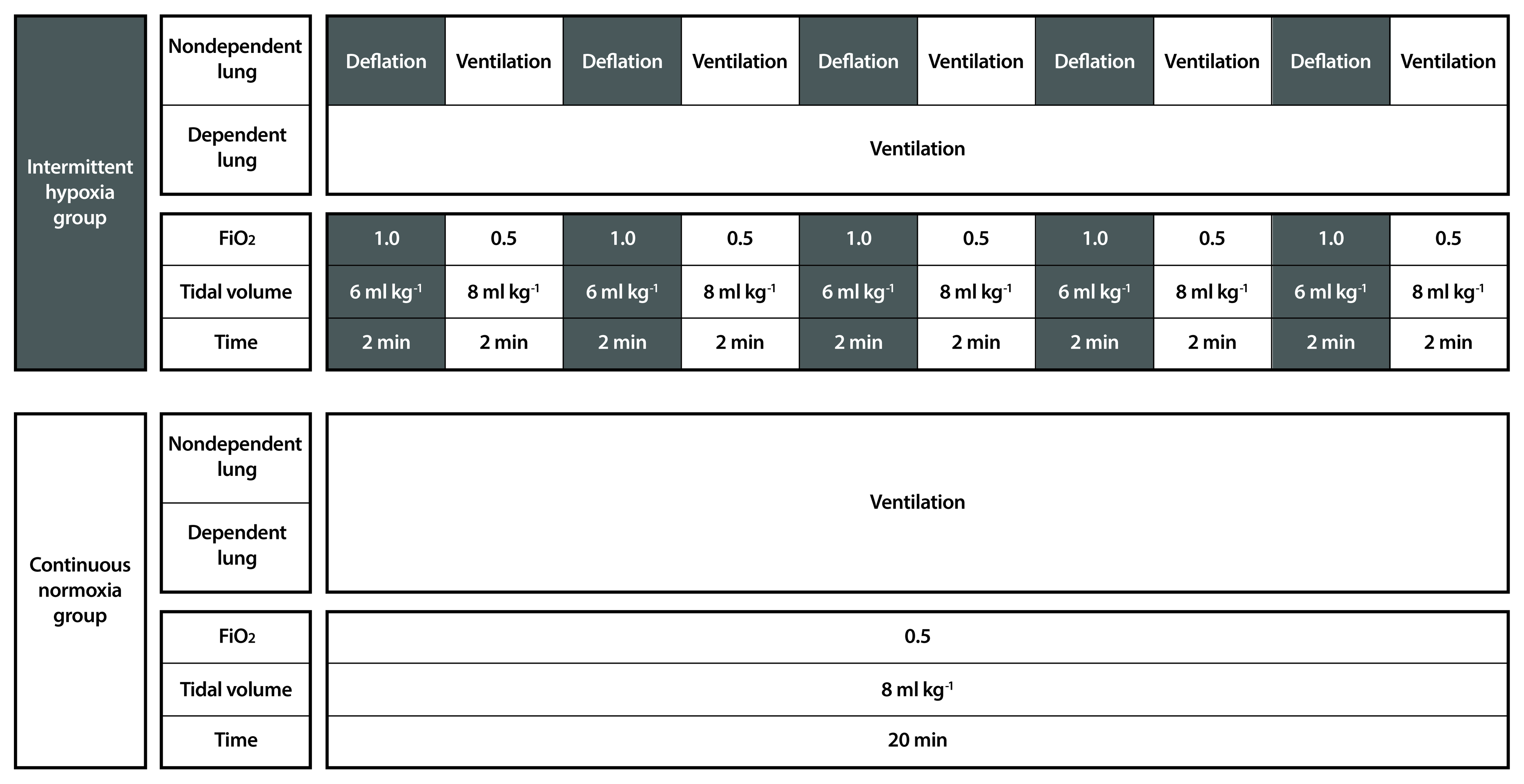

Supplement: S1 Fig — (TIF) [file pone.0249880.s005.tif]
